# Clinical effect of uterine artery embolization combined with curettage in the treatment of cesarean scar pregnancy

**DOI:** 10.12669/pjms.42.6.14466

**Published:** 2026-06

**Authors:** Tao Zhang, Jihong Zhu, Miao Li, Lining Qi, Qiumei Ti, Huiyan Li

**Affiliations:** 1 Tao Zhang, Department of Obstetrics and Gynecology, Baoding No.1 Central Hospital, Baoding 071000, Hebei, China; 2 Jihong Zhu, Department of Obstetrics and Gynecology, Baoding No.1 Central Hospital, Baoding 071000, Hebei, China; 3 Miao Li, Department of Obstetrics and Gynecology, Baoding No.1 Central Hospital, Baoding 071000, Hebei, China; 4 Lining Qi, Department of Obstetrics and Gynecology, Baoding No.1 Central Hospital, Baoding 071000, Hebei, China; 5 Qiumei Ti, Department of Obstetrics and Gynecology, Baoding No.1 Central Hospital, Baoding 071000, Hebei, China; 6 Huiyan Li, Department of Obstetrics and Gynecology, Baoding No.1 Central Hospital, Baoding 071000, Hebei, China

**Keywords:** Curettage, Cesarean section pregnancy, Clinical effect, Uterine artery embolization

## Abstract

**Objective:**

To observe the clinical effect of uterine artery embolization combined with curettage in the treatment of cesarean scar pregnancy(CSP).

**Methodology:**

This was a retrospective study. The subjects of study were 100 patients with CSP from Baoding No.1 Central Hospital between December 2020 and December 2024. Included patients were randomly divided into two groups, with 50 cases in each group. Patients in the study group received uterine artery embolization combined with curettage, while those in the control group were given routine ultrasound-guided curettage. Compared intraoperative bleeding volume, length of stay in the hospital, β-hcG recovery time, and vaginal bleeding time; pre- and post-treatment levels of stress indicators such as adrenocorticotropic hormone(ACTH), cortisol(Cor), and aldosterone(ALD); pre- and post-treatment scores of anxiety and depression, and the incidence of complications between groups.

**Results:**

The clinical response rate of the study group and the control group was 100% and 90%, respectively(p=0.02). The length of stay in the hospital, β-hcG recovery time and vaginal bleeding time were significantly shorter, while the intraoperative bleeding volume was significantly lower in the study group than those of the control group(all p=0.00). Post-treatment ACTH, Cor and ALD levels in the study group were lower than those in the control group(all p=0.00). Moreover, post-treatment SAS and SDS scores of the study group were significantly reduced compared to those of the control group(both p=0.00).

**Conclusion:**

Uterine artery embolization combined with curettage is a safe and effective therapeutic approach for the treatment of CSP.

## INTRODUCTION

Cesarean scar pregnancy (CSP) is a rare form of ectopic pregnancy that is defined as the implantation of subsequent gestational sac at the uterine incision scar of the previous cesarean section. It is a long-term complication of cesarean section,^1^ with increasing incidence in recent decades.^2^ There may be a high risk of uterine rupture when the gestational sac is implanted at this site due to thin muscular layer and poor elasticity at the scar site of the uterus.^3^ Moreover, placental abruption or curettage is prone to massive hemorrhage, producing serious impact on female reproductive function and life.^4^ Clinically, it is advocated that once CSP is diagnosed, pregnancy should be terminated in a timely manner, and the lesion should be removed to reduce complications.^5^ At present, available therapeutic options for CSP include medication and surgical treatment. Medication uses drugs such as thyroxine analogues and thyroid hormones to suppress embryonic growth and hence achieve therapeutic effect, which is suitable for patients with early detection and stable conditions.

However, it is not suitable for all patients, and curettage may be required for treatment in case of severe conditions. Notably, despite definite effect, curettage alone may induce uncontrollable massive hemorrhage intraoperatively or postoperatively. Significantly, uterine artery embolization can block blood supply to the lesion to reduce bleeding, is a feasible procedure for preserving fertility, and has advantages of minimally invasive and safe operation.^6^ Silva B et al.^7^ thought that the optimum therapeutic option for CSP was to achieve a definite therapeutic effect, with the uterus and fertility preserved, and the risk of massive hemorrhage reduced; and recommended a combined therapy for benefiting patients more significantly. In this regard, this study was performed by applying uterine artery embolization combined with curettage for treating patients with CSP, with certain results achieved.

## METHODOLOGY

This was a retrospective study. The subjects of study were one hundred patients with CSP from Baoding No.1 Central Hospital between December 2020 to December 2024. Data were retrieved from the hospital information and management system. Collected their various information of all patients. The included patients were randomly divided into two groups, with 50 cases in each group. Patients in the study group received uterine artery embolization combined with curettage, while those in the control group received routine ultrasound-guided curettage. Subjects in the study group and the control group aged 24-35 and 23-37 years old, with an average age of 28.64±3.71 and 29.38±3.60 years old, respectively. There was no significant difference in the comparison of general data between the two groups, suggesting comparability between groups. Table-I.

**Table-I:** Comparison of general data between the study group and the control group (*x̄* ± *s*, n=50).

Indexes	Study group	Control group	t/χ^2^	P
Age (years)	28.64±3.71	29.38±3.60	1.02	0.31
Time of menopause (d)	48.37±4.52	48.59±4.63	0.24	0.81
Gravidity (times)	3.02±0.21	3.08±0.33	1.08	0.28
Number of cesarean sections			0.25	0.62
1 time (%)	41 (82%)	39 (78%)		
≥2 times (%)	9 (18%)	11 (22%)		
Time since last cesarean section (years)	2.67±0.45	2.71±0.13	0.51	0.63
β-hcG (IU/L)	1630.74±25.63	1632.18±24.83	0.29	0.78

P>0.05.

### Ethical approval:

The study was approved by the Institutional Ethics Committee of Baoding No.1 Central Hospital (No.:[2021]024; date: May 27, 2021), and written informed consent was obtained from all participants and their family members.

### Inclusion criteria:

- Women of childbearing age with clear history of cesarean section.
- Women diagnosed with CSP through pelvic enhanced MRI, ultrasound and other imaging examinations.
- Women without history of hormone therapy for 12 weeks prior to surgery.
- Women without contraindications for curettage or interventional treatment.
- Women with normal coagulation function.
- Women with complete clinical data.
- Women whose family members were willing and able to cooperate in completing the study, and those with good treatment compliance.
- Women with written informed consent provided.

### Exclusion criteria:

- Women with severe infectious and endocrine diseases.
- Women who were allergic to contrast agents.
- Women combined with coagulation dysfunction.
- Women with severe organ dysfunction involving the heart, kidney, and liver, etc.
- Women combined with mental illness or severe communication disorders.
- Women with immune system deficiencies.
- Women combined with ectopic pregnancy at other sites.
- Women with history of uterine bleeding.
- Women who voluntarily withdrew from the study midway.

Patients in the control group received routine ultrasound-guided curettage. Patients were instructed to keep a Trendelenburg position for routine disinfection of the external genitalia and local intravenous anesthesia. A speculum was inserted into the vagina to ensure that the cervix was fully exposed to the field of view. After disinfecting the cervix and internal genitalia, the labium anterius was fixed with cervical forceps to probe its depth. Meanwhile, ultrasound-guided dilation of the cervix was performed to explore the condition of the uterine cavity. Under ultrasound, tissue inside the uterine cavity was sucked using a large-sized suction apparatus. After the suction of the most of tissues, a small-sized suction apparatus was used to remove the remaining tissue until the gestational sac at the scar site was completely sucked, and a scraper was applied for scraping when necessary. The removed tissue was delivered for pathological examination. Patients in this group were provided with routine anti-infection treatment after surgery.

Patients in the study group were given uterine artery embolization combined with curettage. Patients were informed to keep their supine position and an X-ray digital subtraction angiography (DSA) system was used intraoperatively. The right inguinal region was aseptically prepped and draped, followed by local anesthesia. A right femoral artery puncture was performed to insert an arterial catheter into the lower abdominal aorta, followed by angiography of bilateral internal iliac artery to observe the distribution of aortic bifurcation and its branches, and the blood supply to the gestational sac, especially bilateral uterine arteries.

Under the guidance of DSA, a 5F catheter or microcatheter was used for super selective catheterization to insert into bilateral uterine arteries, followed by embolization using absorbable gelatin sponge particles (300~500um in diameter). After the observation of satisfied obstruction of uterine arterial blood flow through angiography, the catheter was withdrawn to the iliac artery for angiography. With the confirmation of uterine artery occlusion, the catheter was removed, and the puncture site was wrapped with compression. Patients were informed to keep strict immobilization of the punctured limb for 24 hours. After surgery, attention should be paid to observing the pulsation of foot artery, skin temperature, and color of the lower limb. Curettage was conducted within 24 hours to 72 hours after surgery, and the specific operation process was the same as that of the control group.

### Outcome measures:

### Comparison of therapeutic effect between the two groups of patients:

Significant effective: Complete removal of the gestational tissue, without further surgery during hospitalization, and β-hcG recovered to the normal range; effective: Removal of the gestational tissue, without other surgery during hospitalization, and obvious decrease in β-hcG than that before surgery; and ineffective: uncontrollable and persistent bleeding during or after surgery, with no significant changes in β-hcG than that before surgery, and the presence of indications for re-operation. Overall response rate = (cases with significant effective treatment + cases with effective treatment)/total cases×100%.^8^

### Comparison of postoperative indicators between the two groups of patients:

Intraoperative bleeding volume, length of stay in the hospital, β-hcG recovery time, and vaginal bleeding duration. Comparison of stress indicators between the two groups of patients before and after treatment: Adrenocorticotropic hormone (ACTH), Cortisol (Cor), and aldosterone (ALD) levels.

Comparison of anxiety and depression scores between the two groups of patients before and after treatment: Evaluation of emotional changes of the two groups of patients before and after treatment using the Self-Rating Anxiety Scale(SAS) and Self-Rating Depression Scale(SDS)^9^, respectively, with better emotional states in patients with lower scores.

Comparison of the incidence of perioperative complications, including fever, lower abdominal pain, intrauterine bleeding, pelvic adhesion, decreased amount of menstrual bleeding, etc.

### Statistical analysis:

Data analysis in this study applied SPSS 20.0 software. Measurement data were expressed as (*x̄*+*s*). Inter-group and intra-group comparisons employed two independent sample t-test and paired t-test, respectively; while the comparison of rates was realized by using c^2^ test. P<0.05 was used to indicate the existence of statistically significant difference.

## RESULTS

The clinical response rate was 100% and 90% in the study group and the control group, respectively, which was obviously higher in the former group than that in the latter group, with statistically significant difference (p=0.02, Table-II).

**Table-II:** Comparison of therapeutic effect between the two groups of patients (*x̄* ± *s*, n=50).

Groups	Significant effective	Effective	Ineffective	Overall response rate*
Study group	43	7	0	50 (100%)
Control group	37	8	5	45 (90%)
*χ^2^*				5.26
*p*				0.02

* p<0.05.

The length of stay in the hospital, β-hcG recovery time and vaginal bleeding time were significantly shorter, while the intraoperative bleeding volume was significantly lower in the study group than those of the control group, showing statistically significant differences (all p=0.00, Table-III).

**Table-III:** Comparison of postoperative indicators between the two groups of patients (*x̄* ± *s*, n=50).

Groups	Length of stay in the hospital (d)*	Intraoperative bleeding volume (ml)*	β-hcG recovery time (d)*	Vaginal bleeding duration (d)*
Study group	4.87±1.03	38.25±3.66	15.73±3.07	6.40±1.53
Control group	7.08±1.45	60.30±7.43	23.08±2.61	9.85±2.01
*t*	8.78	18.82	12.90	9.66
*P*	0.00	0.00	0.00	0.00

* p<0.05.

There was no significant difference in pre-treatment ACTH, Cor, and ALD levels between the study group and the control group (p>0.05). While post-treatment ACTH, Cor and ALD levels in the former group were lower than those in the lower group, with statistically significant differences (all p=0.00, Table-IV).

**Table-IV:** Comparison of stress indicators between the two groups of patients before and after treatment (*x̄* ± *s*, n=50).

Indicators		Study group	Control group	t	p
ACTH (ng/L)	Before treatment	35.74±5.03	36.02±4.96	0.28	0.78
	After treatment*	42.26±4.71	47.80±5.33	5.51	0.00
Cor (ng/ml)	Before treatment	117.24±12.46	116.85±12.38	0.16	0.87
	After treatment*	132.61±11.08	141.33±11.17	3.92	0.00
ALD (ng/dL)	Before treatment	13.70±2.17	13.58±2.10	0.23	0.67
	After treatment*	15.84±2.82	22.43±2.54	12.28	0.00

* P<0.05.

No significant difference was found in the comparison of pre-treatment SAS and SDS scores between groups (P>0.05). While post-treatment SAS and SDS scores of the study group were significantly reduced compared to those of the control group, with statistically significant differences (both p=0.00, Table-V). As shown in Table-VI, the incidence of complications was 6% and 20% in the study group and the control group, respectively, which was lower in the former group than that in the latter group (p=0.04).

**Table-V:** Comparison of emotional status between the two groups of patients before and after treatment (*x̄* ± *s*, n=50).

Indicators		Study group*	Control group	t	p
SAS	Before treatment	57.82±4.50	58.13±4.43	0.35	0.73
	After treatment*	44.26±3.20	48.32±4.78	4.93	0.00
SDS	Before treatment	53.57±6.48	54.03±6.82	0.35	0.73
	After treatment*	43.86±6.14	48.07±6.35	3.58	0.00

* p<0.05.

**Table-VI:** Comparison of postoperative complication rates between the two groups of patients (%, n=50).

Groups	Fever	Lower abdominal pain	Intrauterine bleeding	Pelvic adhesion	Decreased amount of menstrual bleeding	Incidence
Study group	0	0	2	0	1	3 (6%)
Control group	0	1	6	2	1	10 (20%)
*χ^2^*						4.33
*P*						0.04

P<0.05.

## DISCUSSION

In this study, the clinical response rate was 100% and 90% in the study group and the control group, respectively (p=0.02); meanwhile, the length of stay in the hospital, β-hcG recovery time and vaginal bleeding time were significantly shorter, while the intraoperative bleeding volume was significantly lower in the study group than those of the control group (p=0.00); and the incidence of complications was 6% in the study group. These results suggest that the proposed combined therapy is effective in treating CSP, with less intraoperative bleeding and shorter length of stay in the hospital, which is more beneficial for postoperative recovery of postpartum women. In terms of the cause for improved therapeutic effect, it can be explained by the synergistic effect of uterine artery embolization combined with curettage to give full play to the advantages of both. Specifically, pre-treatment via uterine artery embolization can block uterine artery blood flow to reduce risks of intraoperative bleeding, postoperative vaginal bleeding, and massive hemorrhage; and the application of curettage jointly can facilitate a complete removal of the residual gestational tissue, which is also one of the reasons for accelerated recovery in patients after surgery. Uterine artery embolization is a minimally invasive surgery commonly used for postpartum hemorrhage, acute massive hemorrhage, and cervical pregnancy-induced massive hemorrhage, with a vital role played in the treatment of CSP recently. The therapeutic principle of uterine artery embolization is to promote trophoblast cell necrosis by blocking uterine artery blood flow through embolization.^10^ The gelatin sponge used during embolization is a biodegradable albumin glue that can retard blood flow velocity, without damage to capillaries. Mechanical embolization of arteries can promote thrombosis to occlude arterial ducts and thus reduce the incidence of massive hemorrhage.^11^ For the treatment of CSP, the application of uterine artery embolization can accelerate embryo death, and effectively control CSP-induced bleeding, which is independent of gestational age.^12^ Significantly, uterine artery embolization combined with curettage can avoid residual embryonic tissue and excessive damage to the endometrium caused by curettage. Cesarean scar pregnancy(CSP) is a common ectopic pregnancy after cesarean section clinically, without typical symptoms at an early stage. Without timely termination of pregnancy, it may cause uterine rupture as the gestational sac grows that may induce massive hemorrhage, hemorrhagic shock, and resection of the uterus, resulting in the loss of fertility and endangering postpartum women’s life.^13^ Clinical treatment of CSP follows principles of removing embryos, controlling bleeding, preserving fertility as much as possible, and reducing complications. The preferred option for CSP is surgery, generally including hysteroscopic electric resection, curettage, etc. The combined therapy got more favor in clinical practice.^14^ Ultrasound-guided curettage is a common and cost-effective choice for terminating early intrauterine pregnancy. However, it may show poor safety and may be accompanied by risks of massive hemorrhage and uterine perforation, especially for those with high baseline β-hcG levels and large lesion volumes, which are prone to uncontrollable massive hemorrhage.^15^

Notably, curettage is an invasive procedure that may cause a certain degree of stress reaction in patients under anesthesia and operation. ACTH, Cor and ALD are stress hormones secreted by the hypothalamus-pituitary-adrenal axis. The three indexes may show increased levels in the presence of stimuli, and participate in physiological processes such as energy metabolism and temperature regulation to mediate stress response. Surgical complications (e.g., pain and bleeding) can also cause immobility in postpartum women to raise their tension and anxiety.^16^ Additionally, when suffering from CSP, patients with a desire to have another child may experience exacerbated physical pain, accompanied by significant reproductive pressure to themselves and their family. Therefore, attention should also paid to patients’ psychological state, in addition to CSP treatment.^17^ In the present study, post-treatment ACTH, Cor and ALD levels were lower in the study group than those in the control group (p=0.00); moreover, post-treatment SAS and SDS scores in the former group were significantly decreased than those in the latter group (p=0.00). These data support that uterine artery embolization combined with curettage can further alleviate the stress response of postpartum women, relieve pain, reduce bleeding, and stabilize patients’ emotional state.

Furthermore, complications such as bleeding during subsequent curettage may be reduced due to the blockage of arterial blood flow through uterine artery embolization. Besides, local necrotic and harmful substances are removed intraoperatively to prevent their entry into the bloodstream, resulting in reduced complications such as fever as well.^18^ In this study, the incidence of complications in the study group was lower than that in the control group (6% vs. 20%; p=0.04). It suggests that the combined therapy can reduce the incidence of surgical complications when treating CSP, which was basically consistent with that reported by Wang S et al.^19^ It may be related to the minimally invasive operation of uterine artery embolization, resulting in less trauma when combined with curettage.^20^

### Limitations:

It includes a small sample size, shorter period of follow-up, and no consideration of long-term therapeutic outcome.

## CONCLUSIONS

In conclusion, uterine artery embolization combined with curettage is a safe and effective therapeutic approach for the treatment of CSP. Compared with curettage alone, it exhibits obvious curative effect that can reduce intraoperative bleeding, shorten the length of stay in the hospital, accelerate postoperative recovery, lower the incidence of complications, alleviate stress reactions, and improve patients’ emotional state.

### Recommendations:

In order to benefit more patients, our future research based on expanded sample size and extended follow-up will be performed to continuously evaluate the effect of this treatment plan more objectively.

### Authors’ Contributions:

**TZ** and **JZ:** Designed and did statistical analysis & editing of manuscript, is responsible for integrity of research.

**ML** and **LQ:** Performed the statistical analysis and participated in its design.

**QT** and **HL:** Participated in acquisition, analysis, or interpretation of data and draft the manuscript.

All authors have read and approved the final manuscript.
